# Suppression of Motor Sequence Learning and Execution Through Anodal Cerebellar Transcranial Electrical Stimulation

**DOI:** 10.1007/s12311-022-01487-0

**Published:** 2022-10-14

**Authors:** Angela Voegtle, Clara Terlutter, Katharina Nikolai, Amr Farahat, Hermann Hinrichs, Catherine M. Sweeney-Reed

**Affiliations:** 1 Department of Neurology, Neurocybernetics and Rehabilitation, Otto von Guericke University Magdeburg, Leipziger Str. 44, 39120 Magdeburg, Germany; 2 Ernst Strüngmann Institute for Neuroscience in Cooperation With Max Planck Society, Deutschordenstr. 46, 60528 Frankfurt, Frankfurt am Main, Germany; 3 Department of Behavioral Neurology, Leibniz Institute for Neurobiology, Brenneckestr. 6, 39118 Magdeburg, Germany; 4 Department of Neurology, Otto von Guericke University Magdeburg, Leipziger Str. 44, 39120 Magdeburg, Germany; 5 Center for Behavioral Brain Sciences - CBBS, Otto von Guericke University Magdeburg, Universitätsplatz 2, 39106 Magdeburg, Germany

**Keywords:** Motor sequence learning, Cerebellum, Primary motor cortex, Transcranial direct current stimulation, Serial reaction time task, Motor inhibition

## Abstract

Cerebellum (CB) and primary motor cortex (M1) have been associated with motor learning, with different putative roles. Modulation of task performance through application of transcranial direct current stimulation (TDCS) to brain structures provides causal evidence for their engagement in the task. Studies evaluating and comparing TDCS to these structures have provided conflicting results, however, likely due to varying paradigms and stimulation parameters. Here we applied TDCS to CB and M1 within the same experimental design, to enable direct comparison of their roles in motor sequence learning. We examined the effects of anodal TDCS during motor sequence learning in 60 healthy participants, randomly allocated to CB-TDCS, M1-TDCS, or Sham stimulation groups during a serial reaction time task. Key to the design was an equal number of repeated and random sequences. Reaction times (RTs) to implicitly learned and random sequences were compared between groups using ANOVAs and post hoc *t*-tests. A speed–accuracy trade-off was excluded by analogous analysis of accuracy scores. An interaction was observed between whether responses were to learned or random sequences and the stimulation group. Post hoc analyses revealed a preferential slowing of RTs to implicitly learned sequences in the group receiving CB-TDCS. Our findings provide evidence that CB function can be modulated through transcranial application of a weak electrical current, that the CB and M1 cortex perform separable functions in the task, and that the CB plays a specific role in motor sequence learning during implicit motor sequence learning.

**Supplementary Information:**

The online version contains supplementary material available at 10.1007/s12311-022-01487-0.

## Introduction

Motor sequence learning, a type of procedural memory, is fundamental to daily living. It enables automation of frequently repeated activities, such as typing, cycling, or playing a musical instrument. Motor skills acquired through extensive repetition can subsequently be performed effortlessly [1, 2], freeing us to act consciously in our environment. Indeed, errors are more common when an implicitly learned sequence is performed consciously rather than intuitively [3]. Understanding the neural basis of motor learning is required to inform new treatment approaches for conditions in which motor learning is impaired, such as in cerebellar ataxia, stroke, or tumour [4, 5]. Motor sequence learning is also critical in rehabilitation, when new motor skills can compensate for the loss of function. Anatomical axonal tracing, brain stimulation, positron emission tomography, and resting state functional connectivity studies have revealed neural circuitry including the cerebellum (CB), basal ganglia, thalamus, and motor cortex [6–11], and imaging and electrophysiological studies have suggested that these circuits underpin motor learning [12–15].

Anodal transcranial direct current stimulation (TDCS) is a non-invasive approach to modulating neural plasticity by lowering the neuronal firing threshold. It has been investigated as a means of modulating motor learning when delivered to CB or primary motor cortex (M1) [16–20]. Modulation of motor learning performance through electrical stimulation would support a causative role for these structures [21]. Studies investigating the impact of CB- and M1-TDCS on motor learning have so far led to conflicting results, however. Anodal CB-TDCS has been reported to produce functional impairment [22–24], enhancement [18, 25, 26], and mixed findings [27]. Analogous effects to electrical stimulation have recently been shown using optogenetic stimulation of the ventral tegmental area in rodents. This technique enables specific modulation of activity at a cellular level [28]. Purkinje cells provide the only output from the cerebellar cortex, synapsing with the deep cerebellar nuclei, and are deemed to play a crucial role in motor timing and learning [29]. Optogenetic Purkinje cell stimulation has resulted in seizure reduction, an inhibitory effect, in rodent temporal lobe epilepsy models, but only when midline cells were stimulated, underlining the importance of the precise location of stimulation application [30, 31]. Similarly, to anodal CB-TDCS, anodal M1-TDCS studies also report both detrimental [19] and enhancing effects [16] on motor learning, as well as a later consolidation effect only [32] or no effect [33–35]. The variable findings could reflect a multitude of differences between study designs, stimulation parameters, including precise stimulation location, and types of motor learning investigated, including not only implicit motor sequence learning but also explicit motor sequence learning, motor adaptation, and classical conditioning.

Here we focus specifically on implicit motor sequence learning, which is frequently assessed using the serial reaction time task (SRTT) [36]. The SRTT involves responding to sequences of stimuli, in which a particular sequence recurs, unknown to the participant, and is thus implicitly learned, as indicated by faster RTs to the recurring than to a random sequence. The contrast with responses to a random series is important, as performance improvement with practice, regardless of sequence type, should be differentiated from motor sequence learning [23]. CB-TDCS has been shown both to enhance [26, 27, 37] as well as impair [23] motor sequence learning in a SRTT. Jongkees and colleagues [23] reported an initial RT slowing during CB-TDCS compared with sham stimulation (Sham), affecting both learned and random sequence types, which they interpreted as reflecting an enhancement of cerebellar inhibitory effects on the motor cortex. The slowing, which particularly affected the learned sequence, was observed only 24 h later. They suggested increasing statistical power through including the same number of learned as random sequences. In contrast, Ehsani and colleagues [26] reported enhanced sequence learning during anodal CB-TDCS, with no RT change but an error reduction, and Ferrucci and colleagues [27] observed faster RTs after CB-TDCS than after Sham. Studies comparing M1-TDCS and Sham during the SRTT have reported faster RTs in the learned condition compared to Sham [16], but also no change in SRTT performance in a group of patients with Parkinson’s disease or in a healthy control group [34].

As the type of motor learning, the time point of evaluation relative to stimulation, as well as the stimulation type, parameters, and location contribute to variable findings [38, 39], comparison of the effects of TDCS between studies is limited. Hence, if the roles of CB and M1 during motor learning are to be compared using TDCS modulation, both structures should be stimulated within a single study design.

Studies so far reporting both CB-TDCS and M1-TDCS have employed different motor learning tasks, including motor sequence learning [26, 37], but also adaptive learning [18] and motor control during visual pursuit [40]. Individual performances in motor sequence learning and adaptation are not correlated [41], however, and neither interference nor facilitation are observed when these learning types are combined in a single task [42], suggesting differing underlying processing. The studies reporting motor sequence learning used sequences comprising eight items [26, 37]. Such shorter sequences are considered to enable the evaluation of explicit rather than implicit learning [43]. A further consideration is variation in stimulating electrode placement, with both bilateral [27] and ipsilateral stimulation [26, 37] reported.

To enable a direct comparison of the roles of the CB and M1 in implicit motor sequence learning, we used the same SRTT paradigm while stimulating each structure and during Sham. Crucially, the same number of repeated and random sequences were presented, enabling a direct comparison of performance, 12-item sequences were used to ensure that learning was implicit, and ipsilateral stimulation was applied to limit modulation of additional structures. Additionally, we took account of the potential susceptibility effect, which refers to the slower RTs initially observed during the learned sequence directly after responding to random sequences and is well-recognised in motor learning paradigms [44]. We hypothesised that separate roles for the CB and M1 in motor sequence learning can be identified through the differing effects of TDCS to these structures on behavioural performance.

## Methods

### Participants

Sixty healthy, right-handed participants aged 19–34 (M = 26.20, SD = 3.32; 35 females) were recruited. Exclusion criteria were: an IQ < 85, assessed with the KAI Short-From Intelligence Test [45]; history of epilepsy or other neurological disorders; significant alcohol (defined as exceeding the recommended limit of 14 units per week), recreational drug or medication abuse, and other clinical or psychiatric disturbances; metallic implants; or previous participation in a stimulation study, all based on self-report.

Each participant was recruited for a single session, and randomly allocated to CB-TDCS (*N* = 20), M1-TDCS (*N* = 20) or Sham (*N* = 20). The experimental groups did not differ in age (*F*(2,57) = 0.22, *p* = 0.807) or IQ (*F*(2,57) = 1.41, *p* = 0.252).

### TDCS

TDCS was performed in accordance with well-established safety guidelines [46], and the stimulation device (DC-Stimulator Plus, Serial 2049, Version 4.3.00.17, neuroConn, Germany) has safety approval. TDCS has been performed for many decades, and no long-term adverse effects have been identified. Standard 7 × 5 cm rubber electrodes were used. Care was taken to ensure the sponge pads covering the electrodes (7 × 5 cm; neuroConn, Germany) were adequately soaked in 0.9% saline solution, through placement in a receptacle of the solution. On removal, they were saturated with the solution and were placed on the head once dripping had ceased. Potential side-effects were evaluated using a questionnaire issued after the session to address acute symptoms during the stimulation, with items covering tiredness, prickling, itching, headache, and nausea.

For CB-TDCS, we applied the most commonly used montage, with the anode over the right CB, 1–2 cm below the inion and 3–4 cm to the right (review [47]). For M1-TDCS, the anode was placed over C3 according to the international 10–20 electrode placement system. An extracephalic location over the ipsilateral deltoid muscle was chosen for the reference electrode to avoid the potentially confounding effect of cathodal stimulation over another brain region [48, 49]. Cathodal stimulation is deemed to have the opposite effect to anodal stimulation, raising the neuronal firing threshold, thus reducing the likelihood of firing [50]. Participants in the Sham group were randomly assigned to one of the two montages, resulting in an equal distribution. Stimulation in the two experimental groups was applied at 2 mA with a gradual increase over 30 s, lasted for 15 min, and was then reduced again over 30 s. Current was applied for 30 s in the Sham group, for blinding to group allocation [51], as is customary, because tingling side-effects are usually only perceived in the first few seconds of stimulation.

### SRTT Paradigm

**Fig. 1:**
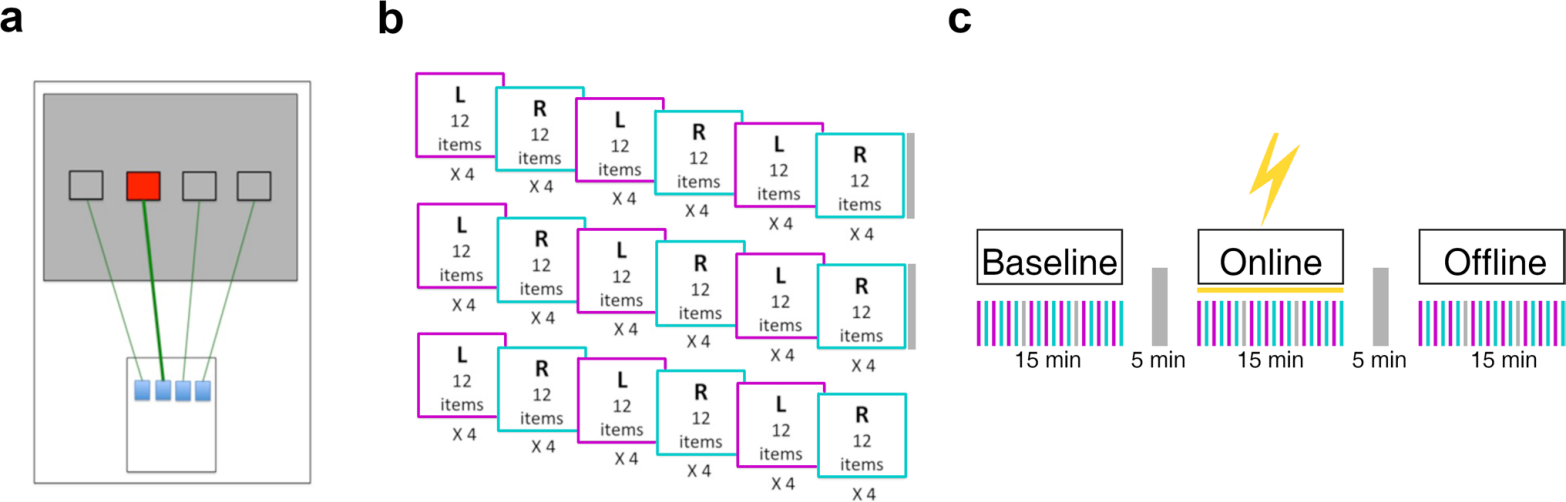
**a** Experimental setup. The participants were asked to place four fingers (index to little finger) on the four keys and press the key corresponding spatially with the square on the screen that turned red as quickly and accurately as possible. **b** Experimental paradigm. A phase consisted of nine learned and nine random blocks in alternating order. A block comprised four sequences of twelve items. After three blocks of each sequence type (learned and random), a short break was taken. **c** Experimental procedure. The experiment had three phases: baseline, online, and offline, each separted by a 5-min break. During the online phase, transcranial direct current was applied

Participants were instructed to press buttons corresponding to the location of a red square as quickly and accurately as possible (Fig. 1a), with the responses made using the four fingers of their dominant right hand. A 12-item sequence (locations: 1–3-2–1-4–1-2–3-1–3-2–4) was used, because participants reported explicit learning with shorter sequences [43]. Although on questioning, some participants suspected a recurring pattern with 12 locations, most recalled 3 or fewer, and none recalled over 4 locations, suggesting implicit rather than explicit learning [52], with implicit motor learning network activation. Each block comprised four repetitions of the 12-item learned sequence or a 48-item random sequence. After three blocks of each type (learned and random), a short break of 30 s was taken (Fig. 1b). In each phase of the experiment, alternating runs of nine learned and nine random blocks were performed, with separate phases completed before (baseline), during (online), and after stimulation (offline) (Fig. 1c). The number of presentations of learned and random sequences, and thereby the statistical power for each sequence type, was equal. Each phase lasted 15 min, and the phases were separated by 5-min breaks.

### Analyses and Statistics

We initially examined a potential effect on RTs of changing between sequence types. We then compared baseline performance between the groups and evaluated whether motor learning took place and was reflected in faster RTs during implicitly learned sequences. Dependencies were then sought between stimulation group and sequence type online and offline. Finally, we examined whether there was a speed–accuracy trade-off. Following evaluation of whether the required assumptions were fulfilled, analyses of variance (ANOVAs) were applied. Greenhouse–Geisser corrections were applied, where Mauchly sphericity was violated with epsilon under 0.75, and Huynh–Feldt corrections were applied when epsilon was greater than 0.75. Bonferroni corrections were applied to the post hoc *T*-tests.

#### Susceptibility Effect

We investigated whether there was a susceptibility effect on RTs to learned sequences due to the change from random to learned blocks [44]. A two-way ANOVA was performed with the within-subject factors *sequence type* (learned, random) and *sequence number* (1 to 4) for the baseline phase. Mean RTs over each sequence number and across the groups were evaluated.

#### Evaluation of Paradigm for Reflecting Motor Sequence Learning and Baseline Performance

We then evaluated whether the paradigm, with our chosen parameters, enabled detection of motor sequence learning and whether the random allocation of partipants to the stimulation groups resulted in similar performance levels before stimulation was applied. We used the RTs of the last sequence for each block, for learned and for random sequences separately for each group at baseline, and performed a three-way ANOVA with the within-subject factors *sequence type* (learned, random) and *time* (9 blocks) and between-subject factor *stimulation group* (CB, M1, Sham). Variances were equal according to Levene’s test for all variables except for the first learned variable. Nine variables (seven learned and two random) were not normally distributed according to the Shapiro–Wilk test. There were three outliers, defined as having mean RTs more than 3 times the interquartile range above the third or below the first quartile. Excluding these participants resulted in a normal distribution of four of the variables which had not previously been normally distributed, but five were still not normally distriubuted, according to the Shapiro–Wilk test. The ANOVA was performed with and without these participants.

To investigate whether a transition from motor sequence learning to the execution of a learned sequence took place before stimulation was applied, or during, or after the stimulation, we performed two-way repeated measures ANOVAs of the RTs pooled across groups during the baseline, the online, and the offline phases, with the factors *sequence type* (learned, random) and *time* (9 blocks).

We then evaluated whether a speed–accuracy trade-off meant that the faster RTs during the learned sequences were at the expense of reduced accuracy. The primary outcome measure of the SRTT is the RT, as the simplicity of the paradigm is expected to enable mostly correct responses, and errors are not likely to reflect the magnitude of learning [53]. Indeed, accuracy measures are often not reported [4, 27, 44]. Moreover, participants were instructed to respond as quickly but also as accurately as possible. However, fast RTs at the expense of accuracy could complicate interpretation, so we examined whether accuracy was lower when RTs were faster. With a maximum of 12 correct responses, the accuracy was near ceiling, and the variables were not normally distributed. As the intention was to exclude a speed–accuracy trade-off, we nonetheless calculated a three-way ANOVA with the within-subject factor *sequence type* (learned, random) and *time* (9 blocks) and between-subject factor *stimulation group* (CB, M1, Sham). Based on the susceptibility effect, the ANOVA was applied to the accuracies of the last sequences of each block, including all participants, as all were included in the ANOVA for RTs. We additionally evaluated the inverse efficiency score (IES) [54], a composite score reflecting RTs and accuracy combined, by dividing the mean correct RTs by the proportions of correct responses [55, 56], to further preclude a speed–accuracy trade-off in our participants.

#### Stimulation: Online and Offline

We performed a four-way ANOVA for RTs, with the within-subject factors *phase* (online, offline), *sequence type* (learned, random), and *time* (9 blocks) and between-subject factor *stimulation group* (CB, M1, Sham). According to the Shapiro–Wilk test, the following variables were not normally distributed: 4 variables for learned sequences during and 5 after stimulation, and 3 variables for random sequences during and 5 after stimulation. Five outliers, with mean RTs more 3 times the interquartile range above the third or below the first quartile, were identified. Their exclusion resulted in a reduction of variables that were not normally distributed from 17 to 15 variables. Each outlier was examined individually. Motor learning was demonstrated for each participant with outlying RTs, based on a faster mean RT to learned than random sequences in each phase. The participants were therefore retained for the subsequent analyses. Variances were homogenous according to Levene’s test.

Post hoc analyses were performed when a significant interaction was identified. Two-way repeated measures ANOVAs were applied in the case of significant three-way interactions, followed by one-way ANOVAs and paired *T*-tests, when indicated.

### Side-Effects

Kruskal–Wallis *H* tests with the grouping variable *stimulation group* (CB, M1, Sham) were performed for the ordinal variables *prickling*, *itching,* and *tiredness*. Two post hoc Mann–Whitney tests were performed to test the two active groups (CB, M1) against the control (Sham). The significant *p*-value was adjusted for multiple comparisons, using Bonferroni correction (0.05/3 = 0.0167). Analysis of *nausea* (*N* = 0) and *headache* (*N* = 2) was omitted, due to sparsity of occurrence.

## Results

### Susceptibility Effect

**Fig. 2:**
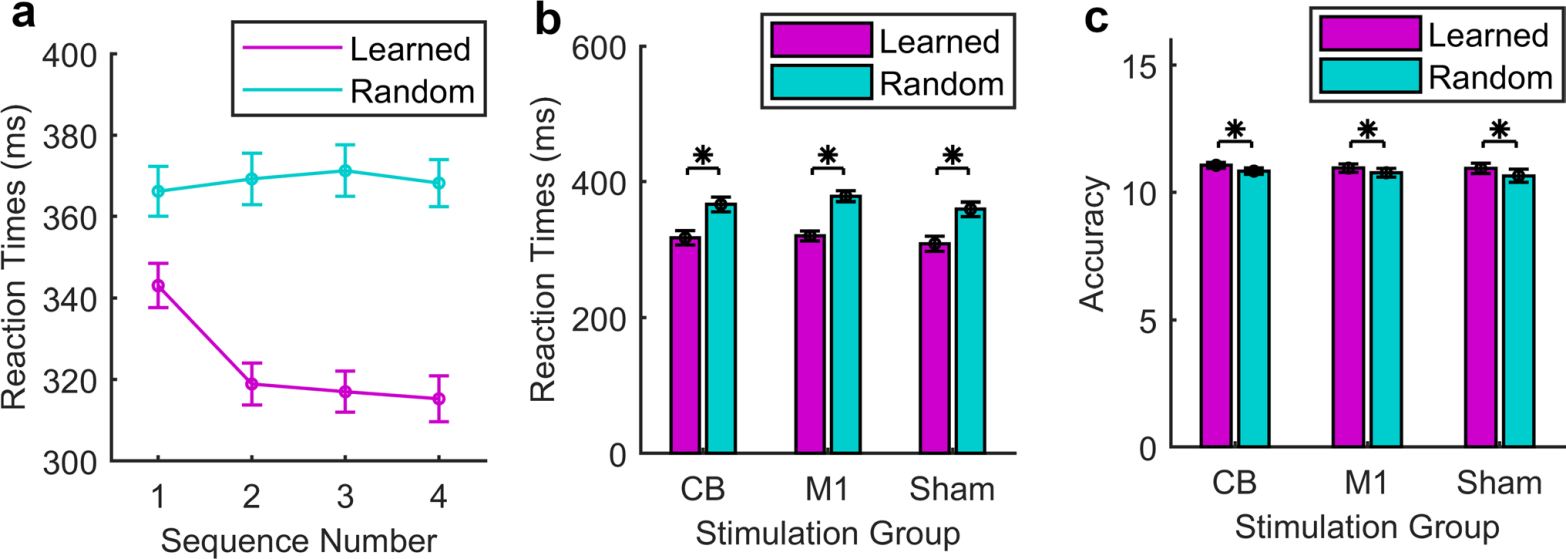
**a** Susceptibility effect. Mean reaction times (RTs) across all groups over the nine blocks at baseline, separately for the four sequences. RTs were faster during the learned sequences. Slower RTs during the first sequence were due to the susceptibility effect. **b** Baseline performance measure for RTs. All three stimulation groups showed motor sequence learning, with faster RTs during learned than random sequences. Baseline performance did not differ between the groups. **c** Baseline performance measure for accuracy as mean number of correct items in a sequence (maximum 12). No baseline group difference in accuracy. Greater accuracy during learned than random sequences suggested no speed–accuracy trade-off

Main effects of *sequence number* (*F*(2.74,161.42) = 34.35, *p* < 0.001) and *sequence type* (*F*(1,59) = 401.79, *p* < 0.001) and an interaction between *sequence number* and *sequence type* (*F*(2.76,162.98) = 56.62, *p* < 0.001) were observed (Fig. 2a). Post hoc paired *T*-tests were then performed separately for learned sequences, to evaluate a potential simple effect of *sequence number* on RTs. The mean RT for sequence 1 differed significantly from sequences 2, 3 and 4 (*T*(59) = 11.09, *p* < 0.001; *T*(59) = 12.05, *p* < 0.001; *T*(59) = 12.95, *p* < 0.001). Sequences 2 and 4 also differed significantly (*T*(59) = 2.18, *p* = 0.034). The RTs during the learned sequences became faster over each 4-sequence block, with maximum learning demonstrated during sequence 4 (the last sequence of each block before switching back to random sequences). The RTs to random sequences did not differ according to *sequence number* (all *p* > 0.05).

We carried out an analogous ANOVA to evaluate accuracy, in case of a speed–accuracy trade-off. Again, a significant interaction was observed between *sequence type* and *sequence number* (*F*(3,177) = 5.02, *p* = 0.002). We therefore examined the accuracy during learned and random sequences separately. Post hoc paired *T*-tests for learned sequences showed that the mean accuracy did not differ significantly between the sequences (all *p* > 0.05). We concluded that the decreasing RTs were not at the expense of decreasing accuracy. As learning progressed over the course of the sequences, as reflected by decreasing RTs to the learned sequences, we performed the analyses to evaluate the impact of *stimulation group*, *sequence type*, *phase* and *time* based on the values during sequence 4 (the last sequence) of each block.

### Evaluation of Paradigm for Reflecting Motor Sequence Learning and Baseline Performance

A main effect of *sequence type* was observed (*F*(1,57) = 392.96, *p* < 0.001), with faster RTs during learned than random sequences. A main effect of *time* (*F*(8,456) = 3.21, *p* = 0.001) was also observed. Post hoc testing showed significantly faster RTs at time points 7 and 8 compared with time point 2 (*p* = 0.012; *p* = 0.017, respectively). Note that the time points correspond with the blocks. There was no difference according to *stimulation group* (*F*(2,57) = 0.63, *p* = 0.54) (Fig. 2b). A two-way interaction was observed between *time* and *stimulation group* (*F*(15.51) = 2.92, *p* < 0.001) (Fig. 5a). Post hoc tests comparing *stimulation group* pairwise at each of the nine time points showed no significant difference between any pair at any time point. Post hoc tests comparing RTs within each *stimulation group* at each *time* point showed differences in the CB stimulation group between time points 1 and 6 (*p* = 0.025) and in the M1 stimulation group between time points 1 and 8 (*p* = 0.012), 3 and 7 (*p* = 0.046), and 3 and 8 (*p* < 0.001) only. There was no significant three-way interaction or two-way interaction between *sequence type* and *stimulation group* or between *sequence type* and *time*. We performed the ANOVA with and excluding the participants with outlying RTs, and there was no alteration in the findings. Moreover, examining the RTs for these participants separately showed faster RTs during the learned than random sequences, suggesting successful motor sequence learning in these participants. The participants were therefore retained.

**Fig. 3:**
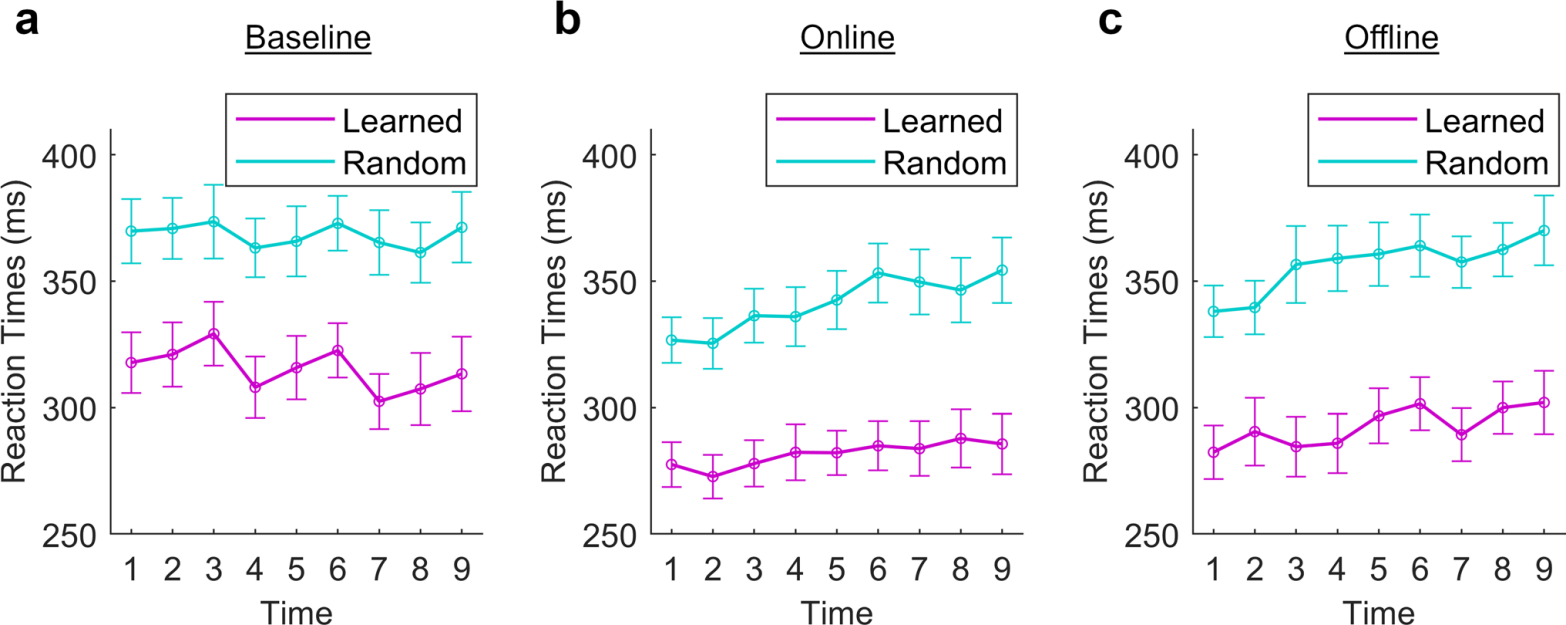
Examination of motor sequence learning and execution. **a** Baseline phase. Improved reaction times (RT) in blocks 4 and 7, after the short breaks, were observed during learned sequences. Progressive motor sequence learning was observed when comparing analogous time points between the breaks (see Fig. 1b). This pattern was not as distinctive for random sequences. **b** Online phase. The RTs to the learned sequences were faster than during the baseline, but no further decrease was observed after block 2, suggesting that the early rapid learning phase ended at this time, and the online phase comprised chiefly execution of an already learned motor sequence, or the retention phase. The break pattern from baseline is also absent, indicating a more solid performance. The slowing of the RTs during the random sequence is likely to reflect fatigue, and the absence of such slowing during the learned sequences may reflect an element of ongoing learning compensating for fatigue during the learned sequences. **c** Offline phase. Slowing of RTs during learned and random sequences suggests a general increase in fatigue

Examining effects of *sequence type* and *time* on RTs during the baseline phase to distinguish between motor sequence learning and execution, main effects of *sequence type* (*F*(1,59) = 391.03, *p* < 0.001 and *time* (*F*(8,472) = 3.02, *p* = 0.003) were observed, but there was no two-way interaction (Fig. 3a). While the RTs to learned sequences generally decreased over time, they increased over the course of blocks, between the breaks, potentially reflecting fatigue. This pattern was less discernable in the RTs to the random sequences and thus potentially reflects ongoing motor sequence learning compensating for fatigue. During the online phase, main effects of *sequence type* (*F*(1,59) = 410.56, *p* < 0.001) and *time* (*F*(7.62,449.59) = 6.49, *p* < 0.001) were seen (Fig. 3b), and there was a trend towards a two-way interaction between *sequence type* and *time* (*F*(8,472) = 1.84, *p* = 0.068). During the offline phase, main effects of *sequence type* (*F*(1,59) = 466.30, *p* < 0.001) and *time* (*F*(6.67,393.25) = 7.27, *p* < 0.001) were seen (Fig. 3c), and there was a two-way interaction between *sequence type* and *time* (*F*(8,472) = 2.04, *p* = 0.041). Post hoc testing showed significantly faster RTs to learned than random sequences at every time point (all *p* < 0.05). RTs to the learned sequences were significantly slower at time point 6 than 1 (*p* = 0.015), but no other comparisons were significant. RTs to the random sequences were significantly slower at time points 4–9 than at time point 1 (*p* = 0.009; *p* = 0.001; *p* = 0.002; *p* = 0.003; *p* = 0.002; *p* < 0.001, respectively) and at time points 6–9 than at time point 2 (*p* = 0.004; *p* = 0.003; *p* = 0.006; *p* = 0.001, respectively).

A main effect of *sequence type* on accuracy was detected (*F*(1,57) = 14.14, *p* < 0.001), with a higher accuracy for learned sequences compared to random sequences, as well as *time* (*F*(8,456) = 3.8, *p* < 0.001), post hoc testing showing a difference between block 2 and block 9 with higher values in accuracy in the earlier block (*T*(59) = 3.69, *p* < 0.001). There was no main effect of *stimulation group* (*F*(2,57) = 0.20, *p* = 0.817) (Fig. 2c). Although a significant interaction between *time* and *stimulation group* (*F*(16) = 1.68, *p* = 0.047) was observed, the groups did not differ between each other at any time point. Differences, however, were observed within each group at different time points, indicating learning over time.

As the accuracy was greater during learned than random sequences, there was deemed to be no speed–accuracy trade-off. This was corroborated by the IES: A main effect of *sequence type* was detected (*F*(1,57) = 184.62, *p* < 0.001). Smaller values for learned than random sequences showed that performance was better during learned sequences. No main effects of *time* (*F*(5.17,294.74) = 2.07, *p* = 0.067 or *stimulation group* (*F*(2,57) = 0.70, *p* = 0.50) were detected. Note that the main effect of *time* became a trend after correction for violation of Mauchly’s sphericity. The trend was towards improved performance over time. However, there was a significant interaction between *time* and *stimulation group* (*F*(10.34) = 2.71, *p* = 0.003). Post hoc testing showed that the groups did not differ between each other at any time point. Any differences were due to changes within a group, with only 1 of 36 comparions, from time point 1 to 8 in the Sham group, being significant (*p* = 0.034).

We concluded that motor sequence learning could be detected using the paradigm with the chosen parameters and that the performance did not differ between the groups prior to stimulation.

### Stimulation: Online and Offline

**Fig. 4:**
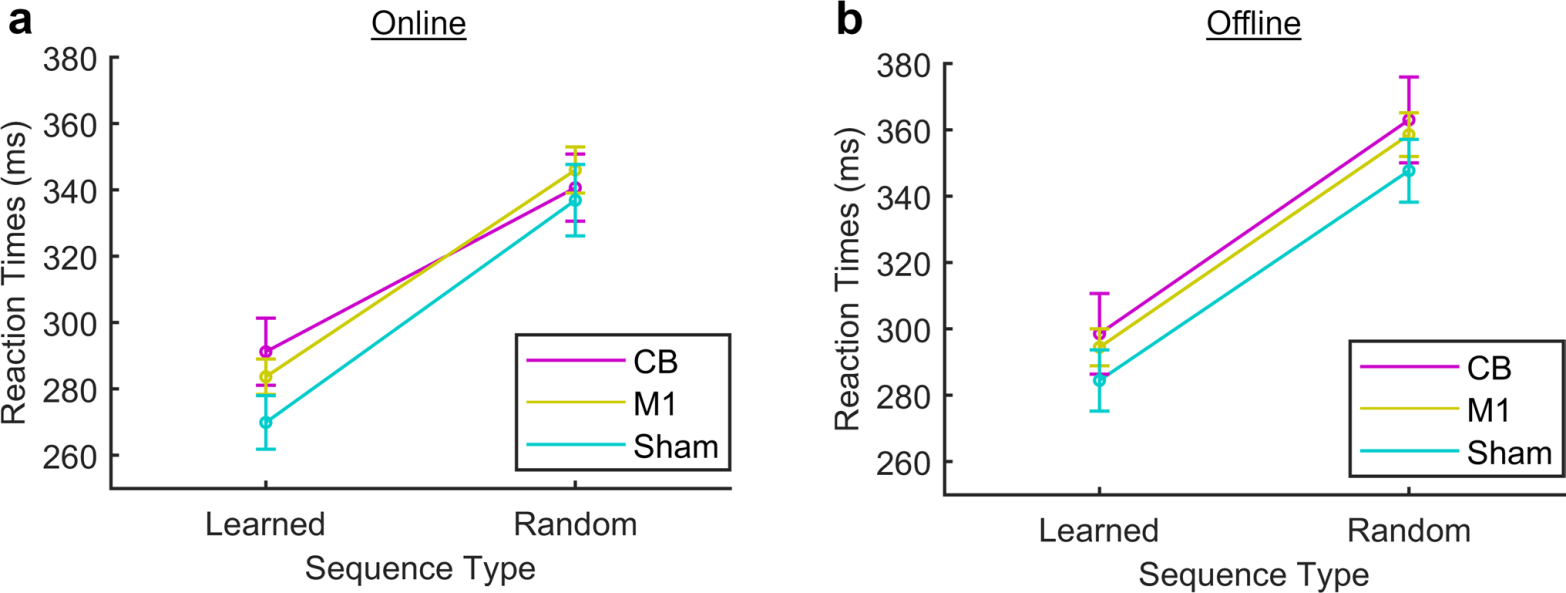
**a** Interaction between the stimulation group and sequence type. RTs were faster during learned than random sequences. **a** Online phase. A significant interaction was observed between *stimulation group* and *sequence type*. **b** Offline phase. No significant interaction

**Fig. 5:**
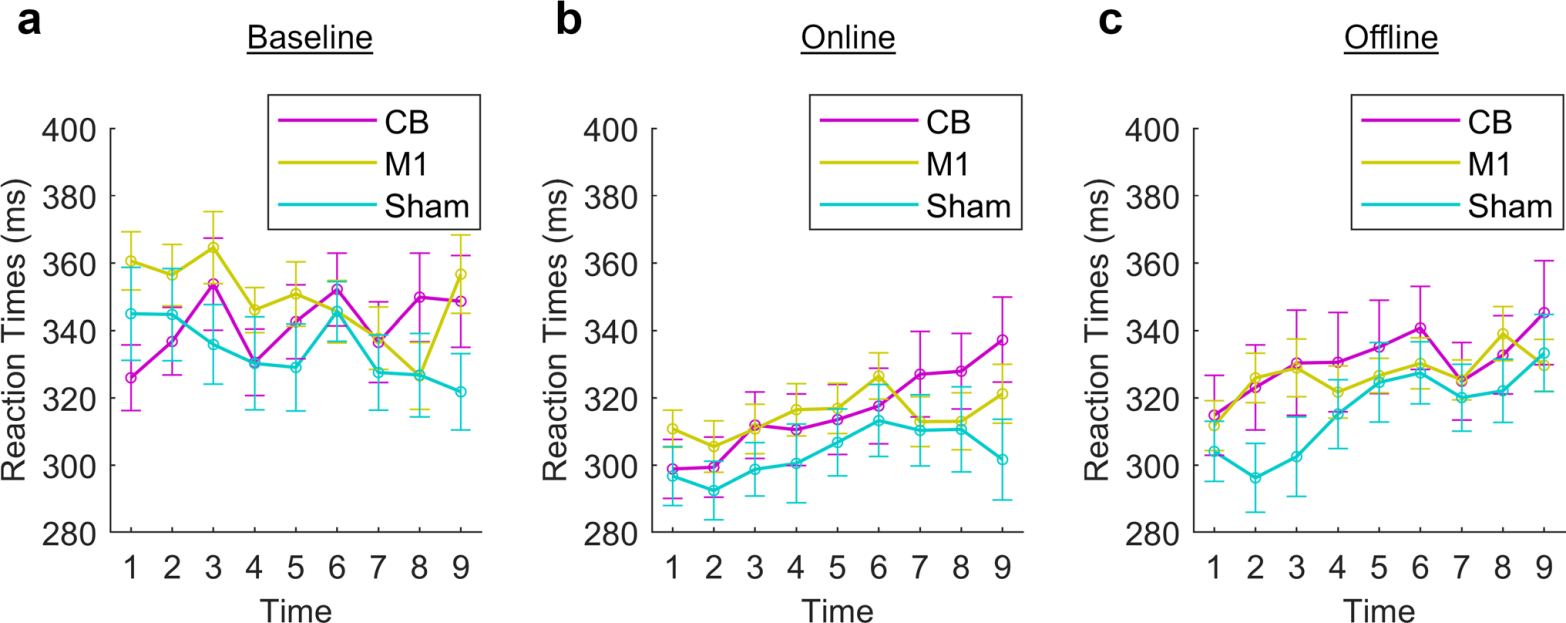
Reaction times during each phase, across learned and random sequences. **a** Baseline phase. Significant interaction between *stimulation group* and *time*. **b** Online phase. Trend towards an interaction between *stimulation* *group* and *time*. **c** Offline phase. No interaction

No significant 4-way interaction was observed, but two significant three-way interactions and three significant main effects were identified. Although the presence of an interaction limits the interpretability of main effects, we chose to report both, as the main effects provide additional information regarding the experiment as a whole. Main effects of *phase* (*F*(1,57) = 38.93, *p* < 0.001), *sequence type* (*F*(1,57) = 596.29, *p* < 0.001), and *time* (*F*(5.85,333.70) = 12.26, *p* < 0.001) were identified. Post hoc testing showed significantly faster RTs during the online than the offline phase (*p* < 0.001), significantly faster RTs during the learned than the random sequences (*p* < 0.001), and significantly slower RTs at time points 5, 6, 7, 8, and 9 than at time point 1 or 2 and at time point 9 than time point 3 or 4 (all *p*’s < / = 0.001). There was no significant main effect of the between-subject factor *stimulation group* (*F*(2) = 0.68, *p* = 0.51). The significant three-way interactions were between the factors *phase*, *sequence type,* and *stimulation group* (*F*(2) = 3.65, *p* = 0.032) (Fig. 4) and between the factors *phase*, *time,* and *stimulation group* (*F*(2) = 1.91, *p* = 0.018) (Fig. 5). Post hoc two-way ANOVAs were performed following each of the significant three-way interactions.

### Interaction: phase, sequence type, stimulation group

Two-way ANOVAs were carried out for each phase, with the factors *sequence type* and *stimulation group.* The mean RTs were calculated over time for learned and for random sequences for each group.

Online, a main effect of *sequence type* (F(157) = 444.68, *p* < 0.001), with faster RTs during learned than random sequences, and an interaction between *sequence type* and *stimulation group* (*F*(2) = 3.45, *p* = 0.038) were identified (Fig. 4a). The interaction indicates that at least one type of stimulation affects RTs to learned sequences differently to random sequences. Post hoc one-way ANOVAs showed that the difference between RTs to learned and random sequences was significantly less during CB than Sham (*F*(1) = 5.38, *p* = 0.026) or M1 stimulation (*F*(1) = 4.32, *p* = 0.045), while the difference between M1 and Sham was not significant (*F*(1) = 0.45, *p* = 0.51). A post hoc one-way ANOVA comparing RTs to learned sequences between CB and Sham showed a trend towards slower RTs during CB (*F*(1,39) = 2.71, *p* = 0.11), whereas comparing RTs to random sequences showed no difference between CB and Sham (*F*(1,39) = 0.065, *p* = 0.80).

Offline, the two-way ANOVA with the factors *sequence type* and *stimulation group* showed only the main effect of *sequence type* (*F*(157) = 450.73, *p* < 0.001), but the interaction between *sequence type* and *stimulation group* was no longer significant (*F*(2) = 0.015, *p* = 0.99) (Fig. 4b).

The three two-way ANOVAs for each stimulation group, with the factors *phase* and *sequence type* and the two two-way ANOVAs for each sequence type, with the factors *phase* and *stimulation group* did not reveal any further significant interactions.

We then applied an analogous ANOVA to accuracy scores to examine whether the interaction during stimulation resulted from a speed–accuracy trade-off. A main effect of *sequence type* (*F*(1,57) = 19.11, *p* < 0.001) showed that accuracy was greater for learned than random sequences, and there was no significant interaction.

### Interaction: phase, time, stimulation group

Next, we calculated the mean RTs across learned and random sequences for each time point. We then carried out two-way ANOVAs with the factors *time* and *stimulation group* for each phase. Online, a main effect of *time* was detected (*F*(6.66, 379.41) = 6.64, *p* = 0.001) but not of *stimulation group* (*F*(2,57) = 0.68, *p* = 0.51). We observed a trend towards an interaction (*F*(16) = 1.66, *p* = 0.052). A general slowing of RTs was observed, with the interaction indicating that the slowing was dependent on the type of stimulation (Fig. 5b). We therefore applied a one-way ANOVA (*F*(2) = 6.71, *p* < 0.002) to the difference between mean RTs at the beginning and end of the online phase, with the factor *stimulation group*, followed by post hoc *T*-tests. The slowing of RTs was greater in the CB than the Sham (*p* = 0.004) or the M1 groups (*p* = 0.018) but did not differ between the M1 and Sham groups (*p* = 1.00).

Offline, there was no longer a trend towards an interaction between *time* and *stimulation group* (*F*(16) = 1.35, *p* = 0.16) (Fig. 5c). A main effect of *time* (*F*(5.90,336.46) = 7.36, *p* < 0.001) was seen but not of the *stimulation group* (*F*(2) = 0.65, *p* = 0.52).

Two-way repeated measures ANOVAs with the factors *time* and *phase* for each stimulation group showed main effects of *time* and *phase* for each group but no significant interactions. Two-way repeated measures ANOVAs with the factors *stimulation group* and *phase* for 8 of the 9 time points showed no significant interaction (see Supplementary Info.).

Again, we considered the possibility of a speed–accuracy trade-off by applying an analogous ANOVA to accuracy values. No significant main effects or interactions were seen, nor was a trend observed.

### Side-Effects

No serious adverse effects, as defined by Bikson and colleagues [57], occurred during this study.

*Prickling* showed a group difference (*H*(2) = 9.674, *p* = 0.008). The M1 group (24.43) differed in mean ranks compared to Sham (16.58; *U* = 121.5, *p* = 0.022, *r* =  − 0.36), as did CB versus Sham (*U* = 94.0, *p* = 0.003, *r* =  − 0.48). Thereby, the mean ranks for the CB group (25.80) were higher compared to Sham (15.20). The two active groups did not differ (*U* = 173, *p* = 0.461).

*Itching* was analysed analogously. A Kruskal–Wallis *H* test revealed a group difference (*H*(2) = 6.187, *p* = 0.045). Post hoc comparison showed that the CB group (24.13) differed in mean ranks compared to Sham (16.88; *U* = 127.5, *p* = 0.013, *r* =  − 0.39), but M1 (23.18) and Sham (17.83) groups did not differ (*U* = 146.5, *p* = 0.047). The two active stimulation groups did also not differ from each other (*U* = 189.5, *p* = 0.746).

The side-effect *tiredness* did not differ between the groups (*H*(2) = 0.703, *p* = 0.704).

## Discussion

Application of anodal CB-TDCS led to modulation of motor sequence learning performance, as reflected by a slowing of responses during the SRTT in comparison with anodal M1-TDCS or Sham, in a cohort of healthy participants. The effect was primarily observed during active stimulation (online). Moreover, a dissociation was observed between the effects of CB- and M1-TDCS, with slower responses during CB- than M1-TDCS to implicitly learned relative to random sequences.

### Preliminary Analysis

We performed preliminary analyses to: (1) address the potential susceptibility effect; (2) establish whether the paradigm allowed evaluation of motor sequence learning using our chosen parameters; (3) verify that the baseline performance of the participants allocated to the three stimulation groups did not significantly differ; (4) evaluate whether and when there was a transition from motor sequence learning to motor sequence execution; and (5) evaluate whether a speed–accuracy trade-off accounted for reduced RTs during the learned compared with random sequences. A susceptibility effect has been well documented in motor sequence learning studies, with slower RTs on the initial presentation of the learned sequence following random sequences [44] and was also found in our paradigm. Hence, we used the mean RT for the last sequence of each block in our analyses, when the greatest observable learning affect had been achieved, with faster RTs during progression through the repetitions of the learned sequence but not during the random sequences. Successful motor sequence learning was demonstrated at baseline, with significantly faster responses during learned than random sequences. We employed a between-subject design, to avoid potential cross-over effects due to differing prior stimulation or practice time. Baseline performance did not differ between the three participant groups according to RTs or to accuracy. There was an interaction between the *stimulation group* and *time* during the baseline. We consider this interaction as unlikely to explain the subsequent inter-group differences during stimulation, however. The inter-individual variability was greater during the baseline phase in all three groups, and only four of the 36 pairwise comparisons between different time points within the groups were significant. Only one of these comparisons was in the CB stimulation group and reflects particularly fast RTs in the first block in this group rather than a trend towards slowing RTs.

Motor sequence learning is an ongoing process. Once a sequence has been learned, faster RTs to the learned than to a random sequence reflect faster execution of the already learned sequence, as well as ongoing learning through further repetition. An overall slowing of RTs to both sequence types over the course of the experiment is consistent with fatigue. To assess whether there was a clear transition from ongoing learning to execution, we examined RTs to learned and random sequences separately for all three study phases. In all phases, RTs to learned sequences were faster than to random sequences, indicating that motor sequence learning took place early in the experiment, and the effect was maintained. During the baseline phase, RTs to learned sequences slowed over time but were faster following each brief break. The finding is consistent with ongoing motor sequence learning throughout the baseline phase, but with superimposed effects of fatigue while the task was continuously performed. In the next phase (online), RTs were again consistently faster during the learned than random sequence. RTs progressively slowed over time, however, during both sequence types, but more during the random than learned sequence. The slowing is again likely to reflect fatigue. The trend towards an interaction between *sequence type* and *time*, with less RT slowing during the learned than the random sequence, suggests ongoing learning compensating for the fatigue. During the final, offline phase, this interaction was significant, suggesting that more ongoing motor sequence learning took place after than during stimulation. The findings suggest that motor sequence learning took place primarily during the baseline phase but did continue through the experiment. Ongoing slow learning, following rapid early learning, is consistent with proposed theoretical models of motor sequence learning [58]. The subsequent comparison between stimulation types thus reflects both execution of the learned motor sequence and also ongoing motor sequence learning, although ongoing learning occurred to a lesser degree than during the baseline phase.

The faster RTs were not at the expense of accuracy, as accuracy was significantly greater during the learned than the random sequences. Moreover, the composite score, IES, reflecting both RTs and accuracy, indicated that overall performance in our participant group was better during the learned than random sequences. Indeed, the application of a repeated measures ANOVA to the IES yielded analogous findings to the ANOVA applied to RTs. As the paradigm is designed to measure motor sequence learning according to RTs [4, 27, 44, 53] and accuracy levels were close to ceiling, we focussed subsequent analyses on the RTs.

### Modulation of Motor Sequence Learning Through CB-TDCS

The significant interaction between *phase*, *sequence type,* and *stimulation group* suggests differential modulation of motor sequence learning performance, depending on the brain region stimulated and whether stimulation was on- or offline. The significant interaction between *sequence type* and *stimulation group* was seen during but not after stimulation. No such interaction was observed based on accuracy scores, rendering the finding unlikely to reflect a speed–accuracy trade-off. Post hoc tests applied to the difference in RTs during learned and random sequences, comparing the stimulation groups pairwise, revealed a significantly smaller difference in RT between sequence types during CB than during Sham or M1 stimulation, with no difference between M1 and Sham. The difference was driven by a difference in the learned blocks rather than the random blocks, with slower RTs to the learned sequence during CB than Sham. This trend was observed only when examining responses to the learned sequence; no tendency to an RT difference between CB and Sham was seen in responses to random sequences. These findings suggest a specific suppression of motor sequence learning through CB-TDCS.

Our findings are in partial agreement with a recent study reporting impaired response selection during anodal compared to cathodal CB-TDCS and Sham [23]. They observed a general slowing of RTs, which was not specific for learned sequences, whereas we detected significant interactions between CB-TDCS and both M1-TDCS and Sham and whether the sequences were learned or random, with a preferential deterioration in response times during the learned sequence during CB-TDCS. Comparing study designs, however, our approach involved greater learning difficulty since we utilised the effect of recurring interference to disrupt learning of the sequence. We found this approach of alternating the sequence type was sufficient to induce stable and successful motor learning, without an impact on RTs to the repeated sequence. The equal number of learned and random sequences applied here also meant that the statistical power of both sequence types was equal. Our findings suggest that CB-TDCS had a specific impact on motor sequence learning rather than a general effect on motor function. The hypothesis that CB-TDCS selectively impairs online motor sequence acquisition, but not motor execution, has also been supported by a negative effect of anodal CB-TDCS compared to cathodal CB-TDCS on accuracy in an explicit motor learning task [24]. In particular, the gamma frequency band seems to play a key role in motor sequence acquisition, as it has been shown that after gamma transcranial alternating current stimulation over the cerebellum, responses to learned sequences slowed during the stimulation, but not to random trials [59]. The findings could be explained by cerebellar brain inhibition (CBI), in which cerebellar Purkinje cells inhibit M1 via the dentate-thalamo-cortical pathway [60]. This inhibition has been shown in a study using transcranial magnetic stimulation to examine changes in CB and M1 following CB-TDCS, in which CBI was facilitated by anodal and hindered by cathodal TDCS [61]. Our finding that anodal CB-TDCS has a detrimental effect on motor sequence learning fits with the notion that lowering the firing threshold in CB through anodal TDCS would increase its inhibitory action on M1. The reduction in temporal lobe seizures in rodent models following optogenetic stimulation of midline Purkinje cells [31] is also consistent with an inhibitory effect when CB is stimulated. CB stimulation is site specific, however [62]. The connectivity of the brain structure stimulated has a bearing on the effects of stimulation, and the effects of optogenetic stimulation to the CB differ according to whether excitatory or inhibitory projections are activated [30]. This consideration is of importance not only with respect to where over the CB stimulation is applied but also in making comparisons between CB and M1 stimulation. Observation of the opposite effects of cathodal stimulation to CB and M1 under the same experimental conditions would provide support for the current findings. We note, however, that while anodal current stimulation over the CB has been shown to reduce motor cortex excitability by increasing the excitability of inhibitory Purkinje cells [63], changing current directionality to cathodal current stimulation has also been found to be more complex than a general reversal of the effect [63].

Several factors may explain the contrast between our findings and those of Ehsani et al. [26], who reported enhancement of motor sequence learning, as reflected by a reduced error rate, and also of Liebrand et al. [37] and Ferrucci et al. [27], who reported faster RTs during CB-TDCS. Ehsani et al. [26] and Liebrand et al. [37] employed an eight-item learned sequence. Sequences comprising eight or fewer items have been considered to test explicit memory [43]. Indeed Liebrand et al. [37] reported evidence of explicit memory for the sequence in over half of participants. The lack of inter-group RT difference in the study by Ehsani et al. [26] could also result from using fewer repetitions of the random than of the learned sequence, resulting in statistical power differences for the sequence types [23]. The SRTT paradigm used could also explain the varying findings. Ehsani et al. [26] showed sequences of coloured stimuli at the same spatial location, potentially requiring different processing to that during the spatially based SRTT applied here. The stimulation by Ehsani et al. [26] could have enhanced the association between an abstract stimulus and a response [23]. A further consideration is that Ehsani et al. [26] and Ferrucci et al. [27] reported the difference in RTs between learned and random sequences as opposed to examining them separately. The difference reported could therefore have reflected slower responses to the random sequence rather than faster responses to the learned sequence [23].

### No Modulation of Motor Sequence Learning Through M1-TDCS

Anodal M1-TDCS is deemed to exert an increase in M1 excitability [51] and has been found to improve performance in implicit motor sequence learning [16]. Here, performance was not modulated compared with the Sham group, however. Our finding is in accordance with previous findings employing the SRTT [35] and an explicit sequence learning task [33]. Moreover, a systematic meta-analysis, testing the influence of anodal M1-TDCS, also found no significant changes in RT or accuracy, during or following single-session stimulation [64]. Our finding suggests that either the stimulation did not influence the motor sequence learning network sufficiently to induce changes, or, in our cohort of young, healthy volunteers, a ceiling effect was reached, such that further improvement in motor learning could not be demonstrated, because RTs had already reached the minimum physiologically possible based on nerve conduction times. If this were the case, improved performance, as indexed by faster RTs, would not be possible, and only a stimulation approach leading to interruption of sequence learning, as observed with CB-TDCS, would have the capacity to result in a demonstrable alteration in performance.

Furthermore, emerging evidence shows a limited role for M1 during motor sequence learning [65, 66], emphasising a more prominent role of the cerebellum and possibly the dorsal pre-motor area [43, 66, 67]. The use of large electrodes (35 cm^2^) might account for the findings of Nitsche and colleagues [16], as additional stimulation of the pre-motor area could be more beneficial. Stimulation of additional structures might thus account, to some extent, for the large inter-individual differences in response to M1-TDCS [67].

### Motor Learning Stages and the Effects of CB-TDCS

A division of motor sequence learning into stages is supported both by differing behavioural parameters as well as by the involvement of different brain regions [58, 68]. Rapid improvement, involving the cerebellum, occurs in the early learning stage [69]. This stage was evident here during the baseline phase and also at the beginning of the online phase in the M1 and Sham groups. The rate of learning is reduced in the consolidation phase [70], and based on the RTs in the Sham group, it is likely that this phase was reached from around block 2 in the online phase. While the basal ganglia are increasingly involved during consolidation, the cerebellum continues to play a role, albeit reducing in extent [71]. It is thus plausible that inhibitory effects of CB stimulation might have been greater had the stimulation been administered from the beginning of the experiment. We also note that the RTs during M1 stimulation decreased later in the online phase and speculate that continuing M1 longer into the retention phase could potentially enhance motor sequence learning. Such an effect would be consistent with previous findings showing enhanced consolidation of motor sequence learning through M1-TDCS [72]. The transition from the subsequent consolidation to the retention phase that follows is not clearly defined, however. Indeed, the efficacy of consolidation is reflected in later evaluation of what has been learned [73], and further testing would be required to allow its evaluation. By the retention phase, in which ongoing slow learning continues, cerebellar processing further decreases in importance, while motor cortical regions increasingly engage [58]. Further studies are required to investigate the effects of stimulation at different stages in motor sequence learning.

### Main Effects

We observed main effects of *phase*, *sequence type,* and *time*. RTs were significantly faster during online than offline stimulation. This overall effect, across learned and random sequences, is likely to be due to fatigue over the duration of the experiment. Indeed, such an interpretation fits with the slowing of responses observed over periods of continuous engagement with the task. Moreover, the main effect of *time*, indicating a slowing over the course of the experiment, is in accord with this interpretation. Finally, the main effect of *sequence type* indicates that motor sequence learning was evident using our paradigm throughout the experiment.

### Limitations

Although there was no significant difference between groups before stimulation, inter-individual variation in performance could nonetheless contribute to our findings. A further potential limitation is that participants reported significantly more itching and prickling during active than Sham stimulation. These side-effects may have influenced performance to some degree. However, since the frequency of the side-effects did not differ between CB-TDCS and M1-TDCS, side-effects cannot explain why a detrimental effect on performance only occurred during CB-TDCS. The finding raises the question, however, as to whether the current standard of blinding [74] is sufficient.

## Conclusion

The present study suggests that CB-TDCS results in a temporary impairment of motor sequence learning, which does not persist beyond the stimulation. This effect was not observed during M1-TDCS, which confirms previous studies, showing distinct roles for CB and M1 in the motor learning network, and adds new evidence supporting an inhibitory effect of the cerebellum.

### Supplementary Information

Below is the link to the electronic supplementary material.

Supplementary file1 (DOCX 13.6 KB)
