# Supplementary material for: Suppression of Motor Sequence Learning and Execution Through Anodal Cerebellar Transcranial Electrical Stimulation
Source: Cerebellum. 2022 Oct 14;22(6):1152–65. doi: 10.1007/s12311-022-01487-0 (PMC10657296; doi:10.1007/s12311-022-01487-0)
Supplement: Supplementary file 1 — Supplementary file1 (DOCX 13.6 KB) [file 12311_2022_1487_MOESM1_ESM.docx]

**Supplementary Information**

Two-way repeated measures ANOVAs with the factors *time* and *phase* for each stimulation group showed main effects of *time* and *phase* for each group but no significant interactions. Two-way repeated measures ANOVAs with the factors *stimulation group* and *phase* for 8 of the 9 time points showed no significant interaction. For 7 time points, a main effect of *phase* was observed (T1: F(1,57) = 5.82, p = 0.019; T2: F(1,57) = 14.60, p < 0.001; T3: F(1,57) = 6.93, p = 0.011; T4: F(1,57) = 8.95, p = 0.004; T5: F(1,57) = 8.95, p = 0.004; T6: F(1,57) = 10.76, p = 0.002; T8: F(1,57) = 7.22, p = 0.009). At T7, there was no significant main effect. At T9, there was again a main effect of *phase* (F(1,57) = 10.38, p = 0.002) and a trend towards an interaction (F(2) = 2.46, p = 0.094). The slowing of RTs from during (online) to after (offline) the stimulation was greatest in the Sham group, as the Sham group showed the fastest RTs during stimulation. Post hoc paired *T*-tests comparing RT during with after stimulation showed no significant difference for the CB or M1 groups but a significant slowing for the Sham group after stimulation (T(19) = 3.65, p = 0.002).
